# Diverse Bacterial PKS Sequences Derived From Okadaic Acid-Producing Dinoflagellates

**DOI:** 10.3390/md20080009

**Published:** 2008-05-22

**Authors:** Roberto Perez, Li Liu, Jose Lopez, Tianying An, Kathleen S. Rein

**Affiliations:** 1 Department of Chemistry and Biochemistry, Florida International University, Miami, FL 33199, USA; 2 Oceanographic Center, Nova Southeastern University, Dania Beach FL 33004, USA

**Keywords:** okadaic acid, polyketide, polyketide synthase, biosynthesis, *Roseobacter*

## Abstract

Okadaic acid (OA) and the related dinophysistoxins are isolated from dinoflagellates of the genus *Prorocentrum* and *Dinophysis*. Bacteria of the *Roseobacter* group have been associated with okadaic acid producing dinoflagellates and have been previously implicated in OA production. Analysis of 16S rRNA libraries reveals that *Roseobacter* are the most abundant bacteria associated with OA producing dinoflagellates of the genus *Prorocentrum* and are not found in association with non-toxic dinoflagellates. While some polyketide synthase (PKS) genes form a highly supported *Prorocentrum* clade, most appear to be bacterial, but unrelated to *Roseobacter* or Alpha-Proteobacterial PKSs or those derived from other Alveolates *Karenia brevis* or *Crytosporidium parvum.*

## 1. Introduction

**Figure 1:**
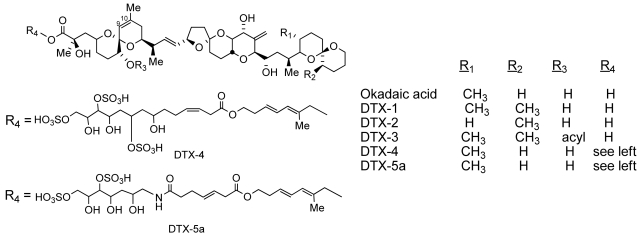
Structures of okadaic acid (OA) and the dinophysistoxins (DTXs).

Dinoflagellates of the genus *Prorocentrum*, most closely related to the genus *Dinophysis*, were first characterized by Ehrenberg in 1833. Species of *Prorocentrum* can be planktonic, benthic, or epiphytic and are distributed globally, most diversely in tropical or sub-tropical marine environments. They are mostly photosynthetic but are known to consume other organisms. The genus is possibly best known for the production of the diarrheic shellfish poisoning (DSP) class of toxins which include okadaic (OA) acid and the dinophysistoxins (DTX) (Figure 1) [1].

DSP has been associated with the consumption of mussels, scallops, or clams tainted with OA and its analogs or derivatives. The acute symptoms of DSP include diarrhea, nausea, vomiting and abdominal pain. Outbreaks have been documented in Japan, Spain, France, Chile, Thailand, New Zealand, Canada, Uruguay, Italy, Ireland, Portugal and Norway [2]. DSP is part of the harmful algae phenomenon which is a risk to health and economies on a global scale [3]. The parent of this group of toxins, OA was first isolated from the sponges, *Halichondria okadai* and *H. melanodocia* [4]. However it was later isolated from laboratory cultures of several dinoflagellates belonging to the genera *Prorocentrum* and *Dinophysis*, including *P. lima*, *P. hoffmannianum, P. concavum, P. maculosum, P. belizeanum, P. faustiae*, *P. arenarium*, *D. acuta* and *D. fortii* [5–13]. In addition to OA, several related polyethers are isolated from these dinoflagellates, including the dinophysis toxins, DTX-1, DTX-2, DTX-3, DTX-4, DTX-5, DTX-5a, DTX-5b acanthifolicin, and multiple diol and sulfated esters of OA and DTXs [14–22]. OA, DTX-1, DTX-2 and acanthifolicin (the 9, 10 episulfide of OA) are inhibitors of protein phosphatases PP-1 and PP-2A [23]. As a result, OA has enjoyed considerable utility as a tool to identify and study processes which are regulated by protein phosphorylation/ dephosporylation.

Stable isotope incorporation experiments have demonstrated that OA and the DTXs are essentially polyketides, although some anomalies in their construction have been identified. [24–26] Polyketides are structurally diverse natural products which share a common biogenic origin. In a fashion analogous to fatty acid biosynthesis, the carbon chain is constructed via the sequential Claisen condensation of small carboxylic acid units catalyzed by a polyketide synthase (PKS). Traditionally, PKS enzymes have been classified as Type I, Type II, or Type III based on the organization of modules and whether they are used iteratively or not [27]. Type I enzymes are analogous to the Type I fatty acid synthases (FAS). The enzymes consist of large multifunctional, modular proteins. Each module contains the functional domains required for a single round of chain extension. This is in contrast to the Type I FAS which is a multi-funtional protein composed of a single module which is used in an iterative fashion. Type II PKSs are multi-enzyme complexes that carry out their functions iteratively. Type III PKS also are iterative enzymes which are responsible for the production of flavonoids, stilbenes, quinoline alkaloids, and acridine alkaloids [28]. Numerous polyketide biosynthetic pathways have been identified from bacteria, plants and fungi. In part due to the large size and complexity of the dinoflagellate genome, no biosynthetic pathway has been identified from this class of organism. However, the structures of the DSP toxins would suggest that they are made by a Type I modular PKS. Indeed, Type I PKS genes have been amplified from cultures of polyketide toxin producing dinoflagellates including *P. lima* [29]. Furthermore, Type I PKS genes have been identified from the related organism *Cryptosporidium parvum* [30].

There is a long standing controversy of whether dinoflagellates actually produce the toxins associated with them or if the compounds have a bacterial origin. Many toxin producing dinoflagellates have not been maintained in the absence of bacteria for an extended period of time. *P. lima* and associated bacteria seem to co-exist on several levels. Bacteria are known to be attached to the surface of the dinoflagellate, residing in the sticky mucus-like phycosphere. However, there is little evidence that *P. lima* contains endosymbiotic or internal bacteria. An ultrastrucural study of the alga has revealed that only “a few percent” of cells contain bacteria-like inclusion bodies residing near the theca of *P. lima*. Additionally, the inclusions are surrounded by a double membrane and contain fibrous material similar to bacterial nucleoids. Interestingly, the inclusions are compartmentalized within the *P. lima* cells by a third membrane [31]. Based on precedence, it seems plausible that these inclusions are indeed bacteria. *P. micans*, for example, has been shown to have bacteria present beneath the theca and attached to the surface [32]. Additionally, intracellular bacteria have been observed in several toxic dinoflagellates, including, *Alexandrium spp*. [33] and *Heterocapsa spp*. [34]. Most interesting, perhaps, is the identification of endosymbiotic bacteria in cells of the DSP toxin producing dinoflagellate, *Dinophysis acuminata* [35].

Extra-cellular bacteria, free-living or attached to *P. lima*, have shown interesting characteristics. As is common in marine environments, they are usually Gram negative Alpha-Proteobacteria. However, the *P. lima* associated bacteria are quite large (2 μm), an unusual occurrence in marine systems. This is indicative of a nutrient rich environment and high metabolic activity [31]. Bacteria from *P. lima* seem to be mostly of the *Roseobacter* genus, although this may be due to selection by culturing or molecular techniques. Regardless, there is at least one reported instance of three bacterial strains producing OA. The bacteria were identified as *Roseobacter algicola*, *Roseobacter denitrificans*, and *Roseobacter litoralis* [36]. Unfortunately, there is no mention of extraction or testing methods which would allow for repetition of the experiments. The bacteria were later shown to be non-toxic by brine shrimp assay. Extracts of the *P. lima* cultures from which the bacteria were isolated were determined to be toxic to brine shrimp indicating that the bacteria may not be producing toxin or may lose toxicity upon culture [37].

The aim of this work was to assess both OA producing and non-producing species of the genus *Prorocentrum* for the presence of PKS encoding genes. Presumably OA producing dinoflagellates would have the same or similar polyketide synthases. Concurrently, we surveyed the bacteria associated with OA producing and non-producing species of *Prorocentrum* for the presence of common bacteria by sequencing of 16S rRNA libraries.

## 2. Results and Discussion

Cultures of dinoflagellates *P. lima*, two strains of *P. hoffmanianum* (CCMP683 and CCMP2804), *P. rhathymum* (CCMP2933), *P. micans* (CCMP2772) and *P. donghaiense* [38], were screened for the production of OA or DTXs by protein phosphatase inhibition assay and by DSP ELISA (Table 1). As anticipated, *P. lima* and both strains of *P. hoffmannianum* were positive in both assays whereas *P. micans* and *P. donghaiense* were negative in both assays. Unanticipated were the small, but detectable amounts of OA equivalents in *P. rhathymum* by both PP2A inhibition and ELISA. *P. rhathymum* was first described in 1979 by Loeblich [39]. After it’s initial description, the *P. rhathymum* taxonomy was dissolved and reclassified as *P. mexicanum* [40, 41]. After much debate the species has recently been reinstated as a unique organism [42]. Our sequence analysis of the large subunit ribosomal genes agrees with the concept that *P. mexicanum* and *P. rhathymum* are separate species [43]. Nonetheless, neither *P. rhathymum* nor *P. mexicanum* have been reported to produce DSP toxins.

Primers (16SF and 16SR, Table 2) to the bacterial small subunit rRNA gene [37] were used to amplify PCR products from the six strains of *Prorocentrum* under investigation as well as extracellular bacteria separated from *P. lima* cultures by tangential flow filtration (TFF bacteria). Gel-purified PCR amplicons were used to construct 16S libraries resulting in a total of 357 cloned PCR products (48 from each species and *TFF bacteria* and 69 from *P. hoffmanianum* (CCMP2804)). These sequences were aligned and submitted to BLASTN for best match identification. Comparison of 16S libraries reveal that over 35 % of all sequences matched most closely with bacteria of the genus *Roseobacter* (Table 3). Furthermore, bacteria of the genus *Roseobacter* represented over 50 % of all sequences originating from OA producing strains of dinoflagellates and were not identified in the non-toxic species *P. donghaiense* or *P. micans*. The identification of *Roseobacter* in association with *P. lima* is not surprising since several species of *Roseobacter* were originally isolated from *P. lima* cultures [36]. Additionally, some strains of *Roseobacter algicola* have been implicated in DSP toxin production [43]. Interestingly, *R. prionitis* was found to be a major constituent of the bacterial library from *P. lima* but was not found in the TFF bacterial fraction suggesting that it is intimately associated with *P. lima.* In fact, the only bacteria identified which were both associated with the *P. lima* cells and in the bulk media was *R. pelophilus* indicating that the bacterial consortium associated with the phycosphere is significantly different from that in the bulk media.

Recently, genes associated with secondary metabolite biosynthesis, PKSs and non-ribosomal peptide synthetases (NRPS), have been amplified from bacteria of the *Roseobacter* clade and it has been suggested that *Roseobacter* should be considered a potential source of new natural products [44]. The phylogeny of PKS genes amplified from *Roseobacter* were found to be related to the phylogeny based on the analysis of 16S rRNA. This report and the abundance of *Roseobacter* found associated with our toxic dinoflagellate cultures, prompted us to examine the PKS encoding genes from cultures of OA producing dinoflagellates to determine if they are related to each other or to other PKS genes originating from *Roseobacter*. Degenerate primers (PKS4U and PKS5L, Table 2) to the β-ketoacyl synthase domain of PKS genes were used to amplify PCR products from the six strains of *Prorocentrum* under investigation as well as bacteria separated from *P. lima* cultures by tangential flow filtration (TFF bacteria). Gel-purified PCR amplicons were used to construct PKS libraries resulting in a total of 280 cloned PCR products (48 from each species except *P. hoffmanianum* (CCMP683) and *TFF bacteria* which produced 24 each and *P. hoffmanianum* (CCMP2804) which yielded 40). Analysis of the 280 resulting translated sequences by alignment (using DNA Star) and translated BLASTX identified a minimum of 16 unique type I PKS sequences (Table 4). One representative of each unique sequence was re-sequenced in both directions to obtain full length sequences of the PCR products (700 bp). Many highly similar sequences (>96 % identity at the amino acid level) were identified from multiple species. These are indicated in Table 4.

**Figure 3:**
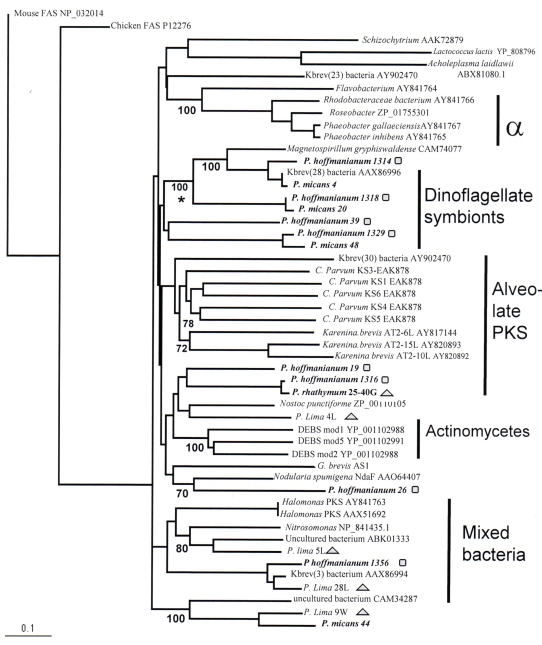
Minimum evolution distance phylogeny generated with the neighbor-joining algorithm and mean pairwise distances of *Prorocentrum*–derived and reference PKS amino acid sequences. GenBank accession numbers are listed next to reference sequences. FAS sequences serve as outgroup. New sequences from this paper are type in boldface. Overall 450 amino acid residues were compared, and of those 81 sites were constant and 269 parsimony informative. After 500 iterations, bootstrap percentages (> 70 %) are shown below each node, while other groups collapsed. Clades are labeled based on the most numerous member sequence. One highly supported *Prorocentrum* dominated clade is marked by an asterisk. Geometric shapes indicate the presence of okadaic acid in the source specimen (squares for relatively high OA levels in *P. lima,* and triangles for relatively low OA levels shown in Table 1). The minimum evolution distance score for this phylogeny was 10.11. The bar on the left shows a scale of 0.10 amino acid residue distance along each branch.

Phylogenetic analysis was performed in order to infer potential organismal sources or relationships for each *Prorocentrum*–derived PKS amino acid sequence, but not absolute phylogenies per se, due to high sequence divergences and lack of optimal outgroups. Reference PKS entries retrieved from GenBank were thus included with new *Prorocentrum* PKS sequences and appear in the minimum evolution phylogeny shown in Figure 3. Similar to previous analyses [45], PKS pairwise distances that were used to construct the phylogeny, ranged widely (2.2 – 75.8 %).The distance tree shows that several *Prorocentrum-*derived PKS sequences appear dispersed with other bacterial PKS sequences in several branches of the tree, although one highly supported *Prorocentrum* symbiont clade includes several PKS sequences from *P. hoffmannianum*, and *P. micans*). No current *Prorocentrum* PKS sequences group with previously characterized *Cryptosporidium parvum* or *Karenina brevis* PKS sequences, supporting a potential prokaryotic source for OA. For example, some *Prorocentrum-*derived sequences grouped with a divergent and heterogeneous group of bacterial PKS sequences, such as *Halomonas*, *Nitrosomonas,* and uncultured bacteria, which were added for reference within the tree. Again, it was interesting that none of the new *Prorocentrum* sequences appear related to *Roseobacter* or Alpha-Proteobacterial sequences. Also interesting is the grouping of OA-associated, and abundant *P. hoffmannianum* (19) and *P. lima* (4L) sequences with *Actinomycetes* Type I PKS sequences.

Outgroup selection did appear to have an influence on tree topology. When vertebrate FAS sequences were excluded from reconstructions, *P. lima* 28L, one of the most frequent PKS-derived sequences in these libraries (Table 4) and *P. hoffmannianum* 1356, often took a basal position near fatty acid synthases (FAS) in the phylogeny. This can be explained by the high amino acid sequence similarities (92 and 99 %, respectively) both share to some fatty acid synthases in BLASTP searches.

Other phylogenetic methods such as maximum parsimony and maximum likelihood, as well as different outgroup sequences (e.g. archaeal PKSs) were also applied to this dataset, but yielded similar groupings and the dispersal of “*Prorocentrum-*derived” PKSs in different parts of the trees.

## 3. Conclusion

At this stage, although there is evidence for a strong association of Alpha-Proteobacterial microbes with multiple *Prorocentrum* species in this study, we cannot easily equate or reconcile the 16S rRNA with the current PKS sequence data. We were expecting to find distinct *Roseobacteria-*like PKS sequences in the recombinant library, which may also be correlated with OA incidence. Of course, this lack could also be attributed to the probable incompleteness of both datasets, as all possible sequences have not been amplified, cloned or sequenced. Alternatively, the predominant PKS library sequences (e.g. PL 28L, PH 19, PL5L, etc) could be associated with some of the various “uncultured” or anonymous bacteria yet to characterized in Table 3. Nonetheless, the PKS phylogenetic analysis indicates that a *Prorocentrum*-specific bacterial PKS clade appears to exist, along with more dispersed *Prorocentrum*-derived sequences. These data leave open the possibility that OA production could stem from one of these bacterial sources. Further experimentation, such as culturing one of these strains for OA production would then constitute a logical next step.

## 4. Experimental Section

### 4.1 Culture Conditions

Cultures were maintained in variety of media: RE (*P. hoffmannianum* CCMP2804, *P. donghaiense,* K (*P. rhathymum* CCMP2933), L1-Si (*P. micans* CCMP2772), f/2-Si (*P. lima and P. hoffmannianum* CCMP683) [46] at 20 – 22 ºC under constant illumination either from Cool White or Grow-Lux wide spectrum lamps. RE medium is based on previously described media such as ASP-M [47] and L1 [48] but differs from most media by the inclusion of high concentrations of biotin and vitamin B12. The RE medium consists of nine separately sterilized solutions (autoclaved unless otherwise noted): (1) 1L artificial seawater containing per liter: 23.38 g NaCl, 4.93 g MgSO_4_•7H_2_O, 4.06 g MgCl_2_•6H_2_O, 0.75 g KCl, 0.2 g KBr, and 12.4 mg H_3_BO_3_; (2) 1 ml RE trace metals containing per liter: 200 mg MnSO_4_•H_2_O, 200 mg Na_2_MoO_4_•2H_2_O, 100 mg ZnSO4•7H2O, 20 mg CuSO4•5H2O, 20 mg CoCl_2_•6H_2_O, 20 mg NiSO4•6H2O, 1.84 mg Na_3_VO_4_, and 1.73 mg Na_2_SeO_3_; (3) 1 ml RE vitamins, filter sterilized, contained per liter: 200 mg vitamin B12 (cyanocobalamine), 200 mg thiamine-HCl, and 100 mg biotin; (4) 1 ml NaNO_3_ (75 g/L); (5) 1 ml NaH2PO4•H2O (5 g/L); (6) 10 ml CaCl2•2H2O (147 g/L); (7) 5 ml Trizma pH 8.1 to 8.2 (279.6 g preset crystals/L); (8) 2 ml NaHCO_3_ (84 g/L); (9) 1 ml FeSO4•7H2O (3.06 g/L freshly prepared and filter sterilized).

### 4.2 Amplification, cloning and sequencing of PKS and 16S genes

#### 4.2.1 DNA Extraction from dinoflagellates

Approximately 1 ml of dinoflagellate culture was pelleted by centrifugation at 10,000 x g for 2 minutes. Genomic DNA (50–100 μl volume) was extracted according to manufacturer’s instructions (Qiagen Bio101 fast DNA kit) using matrix #2 and buffer CLS-Y. The cells were lysed using three cycles of 20 seconds each on the FastPrep FP120 cell disrupter set to speed 4.5. The DNA was quantitated using a μQuant plate reader and diluted to 100 ng/μl for PCR.

#### 4.2.2 Isolation of bacteria from *P. lima*

A non-axenic unialgal culture of *P. lima* was used to isolate bacteria associated with the dinoflagellate. The culture was filtered using glass fiber disks and the filtrate re-filtered using a nylon membrane with a 10 μm pore size in order to remove dinoflagellate cells. The filters were washed with fresh liquid medium (Prov 50) in order to dislodge surface bacteria. The filtrate from the nylon membrane was again passed through a 5 μm pore size membrane. Finally, the bacteria were collected and concentrated (to 50 ml from 4L) using a Millipore lab scale tangential flow filtration (TFF) system equipped with a 0.22 μm filter. The bacterial fraction (named TFF-Bac) was centrifuged at 30,000 x g on a Sorvall super T 21 centrifuge for 1 hour at room temperature. Genomic DNA was extracted from the bacterial pellet after proteinase K digestion [49]. The DNA was screened, and found to be negative, for eukaryotic contamination by small subunit ribosomal RNA (18S) PCR. Additionally, a PKS library of the TFF Bac was prepared to help assess the origin of PKS genes found in the dinoflagellate cultures.

#### 4.2.3 Polymerase Chain Reaction (PCR)

The 50 μl reactions contained 200 ng of DNA, 1X NEB Thermopol buffer, 7 % DMSO, 2.5 mM MgCl_2_, 0.25 nmol each dNTP, 1 μmol each primer, and 1.0U Taq polymerase. PCR reactions were initiated with a denaturing temperature of 95 °C for 2 minutes. followed by the appropriate cycling conditions (28 cycles of 95 °C, 30 seconds; 55 °C, 30 seconds, then 1 cycle of 72 °C, 1 minute 30 seconds for 16S PCR or 40 cycles of 95 °C, 45 seconds; 52 °C, 1 minute 30 seconds, then 1 cycle of 72 °C, 2 minutes for PKS PCR).

#### 4.2.4 Amplicon Purification and Cloning

PCR amplicons were visualized on 1.25 % agarose gels using 1X modified-TAE buffer for gel preparation and electrophoresis. The modified buffer is included in the Montage DNA gel extraction kit (Millipore) and contains a lower concentration of EDTA to prevent interference with enzyme used in further applications. The gels were stained using 2X SYBR gold DNA stain and visualized using a Dark Reader (Claire Chemical Research Inc.). Bands of the anticipated size were excised from the gel and purified using the Montage kit. The DNA was precipitated overnight at −20 °C in 0.1 volumes 3M NaOAc (pH 5.2) and 2 volumes EtOH. Following centrifugation at 20,000 x g for 90 minutes, the DNA was reconstituted in 20 μl of sdH_2_O.

Using the TOPO cloning kit (Invitrogen), 2 μl of the purified DNA was ligated into a pCR 2.1 cloning vector according to the manufacturer’s instructions. One Shot Top 10 chemically competent *E. coli* cells were transformed and incubated for 1 hour in SOC media at 37 °C. LB-agar plates containing 100 μg/ml of ampicillin were covered with 20 μl of 40 mg/ml X-gal (5-bromo-4-chloro-3-indolyl-β-D-galactopyranoside) for blue/white screening of clonal colonies. Each plate was inoculated with 10 μl, 50 μl, or 100 μl of SOC culture and incubated at 37 °C overnight. Colonies (48 per sample), were picked and streaked on an LB-amp plate for purity. From these plates, a single colony was selected and used to inoculate 3ml LB-amp liquid cultures for overnight incubation. Plasmids were purified using the Qiagen QiaPrep miniprep kit and diluted to a final concentration of 250 ng/μl.

#### 4.2.5 Sequencing Reactions

Sequencing reactions were conducted on the Eppendorf mastercycler using a 96-well plate format. Each 10 μl reaction contained 250 ng of plasmid DNA, 1X reaction buffer, 1/16X Big Dye 3.1, and 4.8 pmol of the M13 reverse sequencing primer. The labeled products were amplified using 40 cycles of 95 °C for 15 seconds, 50 °C for 20 seconds, and 60 °C for 4 minutes. Sequencing was performed on an ABI 3730 sequencer (Applied Biosystems, CA). The resulting sequences, on average 500 bp each, were assembled and aligned using the programs SeqMan and MegAlign (Lasergene). Additionally, sequences were submitted to nucleotide BLASTN and translated-BLASTX analysis for identification [50]. One clone from each unique PKS group was sequenced in both directions by MWG biotech, using T3 or T7 primers to provide a full length sequence for the amplicon. 16S sequences were submitted to BLASTN for best match identification. Sequences have been submitted to Genbank. Accession numbers will be provided at the time of publication.

### 4.3 Phylogenetic analysis

Most phylogenetic analyses of PKS sequences followed methods previously described [45, 29]. Several reference PKS amino acid sequences were also previously documented, while the following PKS sequences were retrieved for this study: *Halomonas* AAX51692, *Halomonas* PKS AY851763, *Roseobacter* ZP_01755301, *Phaeobacter inhibens* AY841765, *Nodularia spumigena* AAO64407.1, unknown marine bacterium AAX51693.1, *Nodularia spumigena* AAO64407.1, *Magnetospirillum gryphiswaldense* CAM74077.1, M*ethanosarcina mazei* AAM30566.1, *Picrophilus torridus* AAT43773.1.

Phylogenetic analysis began by aligning sequences using CLUSTALX [51]. For 16S sequences, after manually checking alignments by comparing with known secondary structure models [52], poorly aligned SSU rRNA regions (e.g. high number of gaps or indels) were omitted from further analysis. Alignments were then imported into PAUP (phylogenetic analysis using parsimony) v 4.0b3a [53], which allowed a comparison of various phylogenetic algorithms and substitution models. Due to the large amount of sequence divergence in most PKS datasets, minimum evolutionary tree topologies based on distance models (neighbor-joining), maximum parsimony and maximum likelihood were obtained using heuristic methods. Each reconstructed group was statistically evaluated by bootstrapping with a minimum number of 500 replicates [54, 55]. Most appropriate DNA substitution models for distance algorithms were determined using MODELTEST [56].

### 4.4 Protein Phophatase (PP2A) inhibition assay

Two-hundred mL of each culture were centrifuged at 1020 X *g* for 15 minutes and filtered through a G6 glass fiber filter. The supernatant was passed through a 2g C-18 SPE column, washed with water (30 mL) and eluted with MeOH (50 mL). The MeOH was dried in vacuuo and the residue was resuspended in 1 mL of MeOH. Dilutions of this extract were used for both the PP2A inhibition assay and the okadaic acid ELISA.

The PP2A inhibition assay, modified from Simon [57] and Tubaro [58], was conducted as follows: each sample was prepared in 20 μl of 50 %MeOH in H_2_O and combined with 0.15 U of PP-2A in 20 μl of buffer containing Tris (50 mM, pH: 7.4), Na_2_EDTA (10 mM), MnCl_2_ (10 mM), 1.3 μl BSA (0.1 μg/μl), 0.15 μg Dithiothreitol (DTT) in H_2_O. The sample was then combined with 180 μl of buffer containing 0.5 mg of pNPP, Tris (50 mM, pH: 8.1), MgCl_2_ (4 mM) and MnCl_2_ (0.4 mM) allowed to incubate for 2 hours at room temperature and read using a Bio-Tek Instruments μQuant plate reader scanning at 405 nm. A standard curve was prepared with pure OA, prepared exactly as the samples, using serial dilutions from 20 nM to 0.625 nM OA in 50 % MeOH.

### 4.5 OA/ DTX ELISA

The okadaic acid ELISA was performed according to the manufacturer’s (Abraxis) instruction, using the methanol solution prepared for the PP2A inhibition assay (section 3.4). Serial dilutions were performed in the assay buffer, such that the concentration of OA equivalents was in the quantifiable range.

## Figures and Tables

**Table 1:** Extracellular Concentrations of OA equivalents.

	OA equivalents by PP2A (ppb±sd)	OA equivalents by ELISA(range in ppb)
*P. lima*	235 ± 5	328 ± 6
*P. hoffmanianum*	97 ± 3	81 ± 1
*P. rhathymum*	0.45 ± 0.004	0.57 ± 0.003
*P. micans*	nd^a^	nd^a^
*P. donghaiense*	nd^a^	nd^a^

a Not detectable n = 3for PP2A n = 2 for ELISA

**Table 2:** Sequences of PCR primers.

Name	Sequence (5’→3’)	Product Size
PKS4U	MGIGARGCIYTICARATGGAYCCICARCARMG	700bp
PKS5L	GGRTCNCCIARYTGIGTICCIGTICCRTGIGC	
16SF	GGAGAGTTTGATCATGGCT	1.3kbp
16SR	ACGGYTACCTTGTTACGACTT	

**Table 3:** Results of 16S rRNA libraries indicating the best matches from nucleotide BLAST searches and frequency of occurrence within each library.

Accession number	Nearest BLASTn Match Description	PL	TB	PH1	PH2	PR	PD	PM
DQ167249	*Roseobacter prionitis*	28.5		17		60		
AJ968652	*Roseobacter pelophilus*	28.5	37					
DQ120726	*Roseobacter sp.* 812		6	17				
DQ104407	*Roseobacter sp.* JL-351				49			
DQ659415.1	*Roseobacter* sp. COL2P				8.7			
AJ878874	*Thalassobius mediterraneus*		6	9				
AM420114.1	Uncultured Alpha-Proteobacterium		6	9				
AY162118	*Planctomycete* GMD16E07		6			22.		
AJ889010	*Stappia alba* strain 5OM30		6			5		
AY163576	*Croceibacter atlanticus*					3	51	
EF414083	Uncultured *Bacteroidetes* bacterium					3		3
Y15341	*Rhizobium sp.*	17.5				3		
AY654759	Mucus bacterium 110 from *O. patagonica*	4.5						
AY258089	*Mesorhizobium sp*. DG943	3						
AF441991.1	Uncultured CFB group bacterium clone	3						
EF12346.1	Uncultured Gamma-Proteobacterium clone	3						
DQ446117	Uncultured spirochete clone	3						
AJ227758	*Caulobacter henricii* strain ATCC 15253		9					
AY136121	*Marinobacter* sp. MED106		6					
AY701447	Uncultured *Bacteroidetes* bacterium		6					
AM176885.1	Uncultured bacterium clone SZB60		6					
AY917783	Uncultured bacterium clone 1971b-30		6					
DQ811828	Uncultured Delta-Proteobacterium clone				5.8			
AY162122	MSB				4.3			
AY960750	P*lanctomycete* GMD14H10				3			
AY345437	*Maribacter dokdonensis* strain DSW-9				3			
AY517632	Bacterium K2–12				3			
DQ513013	*Marinobacter flavimaris* strain SW-145				3			
DQ486493	Uncultured bacterium clone FS140-15B-02				3			
EU196324	Gamma-Proteobacterium DG1253				3			
EU148878	*Sphingomonas* sp. NP31			12				
EF106349	Uncultured bacterium clone PP6–13			9				
EF658677	Uncultured planctomycete			9				
AY038570	Uncultured bacterium clone YHSS3			9				
AY682384	Uncultured alpha-Proteobacterium			12				
CP000264	*Kordiimonas gwangyangensis* strain			6				
EF658677.1	GW14–5			6				
AY539822	*Jannaschia* sp. CCS1							
AY258095	Uncultured bacterium clone YHSS3					8.5	34	
EU249979	Gamma-Proteobacterium BT-P-1						4.5	
AY664364	*Sulfitobacter sp*. DG1020						4.5	
AY562560	Uncultured Pseudomonas sp						3	
AM697073	*Pseudoalteromonas sp.* PM02						3	
AY344411.1	Alpha proteobacterium CRA 4C							41
EF123623.1	Uncultured bacterium							17.5
EU107173.1	Unidentified bacterium clone K2-S-32							10.5
EF512127.1	Uncultured *Bacteroidetes* bacterium							8.5
AB255368.1	*Pseudomonas* sp. N9-1							4.5
DQ822527.1	*Stappia alba* strain							4.5
EU196324.1	*Gilvibacter sediminis*							3
EU005335.1	Bacterium QM28							3
AB073564	*Sphingomonas* sp. NP31							3
	Uncultured alpha proteobacterium clone							
	G7–25							
	*Cytophaga sp.* MBIC04693							

*P. lima* (PL), *P. hoffmanianum* (PH1, CCMP683 or PH2, CCMP2804), *P. rhathymum* (PR), *P. micans* (PM), *P.donghaiense* (PD), and TFF bacteria from *P. lima* (TB).

**Table 4:** Alignment results of the PKS libraries

PKS sequence name	Total Occurrence Frequency (%)	Organism Libraries

PL 28L	24	PL, PD, PR, PM,
PH 19	18	CCMP2804
PL 5L	18	CCMP683, TB
PL 4L	11	PL, CCMP683, TB
PL 9W	1	PL, CCMP683, TB
PH 26	0.5	PL, TBCCMP683
PH 39	0.5	CCMP683
PR 25	1	PR, CCMP2804
PM 44	6	PM
PM 4	3	PM
PM 48	2	PM
PM 20	0.5	PM, CCMP2804
PH1316	10	CCMP2804
PH1329	2	CCMP2804
PH1356	1	CCMP2804
PH1314	1	CCMP2804

*P. lima* (PL), *P. hoffmanianum* (CCMP683 or CCMP2804), *P. rhathymum* (PR), *P. micans* (PM), *P. donghaiense* (PD), and TFF bacteria from *P. lima* (TB)
